# Measuring engagement in deliberate self-harm behaviours: psychometric evaluation of six scales

**DOI:** 10.1186/1471-244X-13-4

**Published:** 2013-01-03

**Authors:** Shane Latimer, Tanya Meade, Alan Tennant

**Affiliations:** 1 School of Social Sciences and Psychology, University of Western Sydney, Locked Bag 1797, Penrith, NSW 2751, Australia; 2 Department of Rehabilitation Medicine, Faculty of Medicine and Health, University of Leeds, Gt. George Street, Leeds, LS13EX, UK

**Keywords:** Deliberate self-harm, Self-mutilation, Self-injurious behaviour, Rasch measurement

## Abstract

**Background:**

Engagement in Deliberate Self-Harm (DSH) is commonly measured by behavioural scales comprised of specific methods of self-harm. However, there is a scarcity of information about the degree to which the methods relate to the same DSH construct although such scales are routinely used to provide a DSH total score. This study addresses the shortfall by evaluating the dimensionality of six commonly used behavioural measures of DSH.

**Methods:**

The DSH measures were Self-Injury Questionnaire Treatment Related (SIQTR), Self-Injurious Thoughts and Behaviors Interview (SITBI), Deliberate Self-Harm Inventory (DSHI), Inventory of Statements About Self-Injury (ISAS), Self-Harm Information Form (SHIF) and Self-Harm Inventory (SHI). The behavioural scales contained in each measure were administered to 568 young Australians aged 18 to 30 years (62% university students, 21% mental health patients, and 17% community members). Scale quality was examined against the stringent standards for unidimensional measurement provided by the Rasch model.

**Results:**

According to the stringent post-hoc tests provided by the Rasch measurement model, there is support for the unidimensionality of the items contained within each of the scales. All six scales contained items with differential item functioning, four scales contained items with local response dependency, and one item was grossly misfitting (due to a lack of discrimination).

**Conclusions:**

This study supports the use of behavioural scales to measure a DSH construct, justifies the summing of items to form a total DSH score, informs the hierarchy of DSH methods in each scale, and extends the previous evidence for reliability and external validity (as provided by test developers) to a more complete account of scale quality. Given the overall adequacy of all six scales, clinicians and researchers are recommended to select the scale that best matches their adopted definition of DSH.

## Background

Deliberate self-harm (DSH) (also referred to as self-harm) is a sub-type of self-destructive behaviours [1] that is intentional, direct and immediate in terms of bodily damage [2,3] with a non-fatal outcome [4]. DSH may reflect multiple intentions (i.e., suicidal and non-suicidal) [5] and may serve a range of intrapersonal and interpersonal functions [6].

Currently, there is no comprehensive classification system for describing DSH although several specific definitions have been proposed [7]. Some define DSH as tissue damaging acts performed in the absence of a desire to die (e.g., [8]), a conceptualisation that is mostly called Non-Suicidal Self-Injury (NSSI) [9]. Others define DSH as a broad spectrum of non-fatal self-injury irrespective of degree of type of motivation (e.g., [5]), a conceptualisation that is mostly called Self-Harm (SH) [10,11].

DSH without suicide intent and DSH regardless of intent are the two dominant paradigms in self-harm research and clinical practice [7]. Arguing the relative merits of one approach over the other is challenged by: (a) difficulties in measuring intent [7]; (b) suicidal ideation and intent may accompany superficial, non-life threatening self-harm acts [1]; (c) severe forms of self-harm may lead to potentially fatal outcomes with little or no conscious suicide intent [12]; and (d) suicide and non-suicide related self-harm often co-occurs in the same individual [13].

Notwithstanding the above challenges, the NSSI conceptualisation of DSH is being considered for inclusion in the fifth edition of the Diagnostic and Statistical Manual of Mental Disorders (DSM-5) [14]. The merit of NSSI as an independent disorder is based, in part, on the argument that the methods of DSH most associated with NSSI (viz., mild to moderate forms of visible tissue damage) [15] may form a distinct grouping of behaviours on a DSH continuum [7].

Clinicians and researchers have developed practical strategies to distinguish DSH without suicide intent and DSH regardless of intent. Clinicians generally assess DSH methods first and then clarify intent for each act (e.g., [10]). Researchers orientate participants to respond to questions as suicide or non-suicide related acts by the instructions and item wording in their measurement tools (e.g., [16]).

Both strategies are supported by the large number of published DSH measures that include a behavioural scale comprised of short descriptions of specific methods of self-harm [17]. The endorsement of at least one method of DSH is the accepted procedure for estimating prevalence rates of DSH [18,19]. Counting the number of methods of DSH and summing their frequency over periods of time (commonly a person’s lifetime or over the last 12 months) have been used to examine the relationship between DSH and increased risk of suicide, depression, anxiety and personality disorder [13,20,21]. The formation of total scores (formed by adding the number of methods of DSH or their frequency over a period of time) is based on the premise that (a) the range and frequency of methods is clinically informative, and (b) the various methods included in the counting procedure all relate to the same underlying DSH construct, a property called unidimensionality [22].

It is accepted that clinical assessment is informed by the range and frequency of past DSH methods [23]. There is emerging evidence that the number of different DSH methods may be particularly informative. In cross-sectional research, counts of past methods are more strongly associated with psychopathology as compared to frequency or recency (e.g., [13]). In longitudinal research, counts of past methods are the best predictors of future DSH (e.g., [19]).

However, the unidimensionality of DSH behavioural scales is rarely reported for DSH scales [24], despite this quality being an accepted standard for scale selection [25]. Reasons for not evaluating unidimensionality (when stated by the scale developers) include too few items in the behavioural scales [26] and a lack of acceptance that DSH behaviours are indicators of a DSH latent construct [27]. It should be noted that unidimensionality cannot be assumed from a high estimate of Cronbach’s Alpha [28].

Further investigation of the unidimensionality of DSH behavioural scales is, therefore, warranted to fully inform the selection of DSH scales for clinical and research applications. An appropriate analytic tool is the Rasch measurement model [29]. This model provides strict post-hoc tests of unidimensionality [30] and it is widely used in the development of mental health scales [31]. Moreover, the Rasch model informs the applicability of scales across different populations (item bias) and provides a hierarchy of scale items [32].

The Rasch model can be applied to dichotomous data [29] and polytomous data [33]. The model is therefore applicable to the wide range of response formats (e.g., yes/no for presence of specific methods, rating scales for frequency and recency) included in DSH behavioral scales (see [17]). However, the present study is focused on the application of the Rasch model to lifetime presence of specific methods of DSH (scored 0,1). This is a logical first step as most test developers report scale reliability based on Cronbach’s Alpha (which implies the intention to summate the item scores), and they tend to calculate Cronbach’s Alpha based on dichotomous scores for lifetime presence [24]. It is also a sensible starting point because of the emerging evidence that the number of different DSH methods (as measured by published DSH behavioural scales) may be the best predictor of future DSH [19].

The present study aims to address the shortfall in knowledge about the psychometric properties of published DSH behavioural scales. Specifically it will: (1) evaluate the dimensionality of the DSH methods contained in each scale; (2) examine the presence of item bias for age and gender; (3) inform the hierarchy of items within each scale; and (4) recommend scale selection for researchers and clinicians.

## Method

### Scales

A search of computerised data bases identified 33 tests containing a behavioural scale/sub-scale comprised of specific acts of DSH and published in the English language during the period 1980 to 2010. Of those, 21 tests were deemed the most relevant to this study based on being: (1) appropriate to young adults, (2) not specific to intellectual disability and/or autism, and (3) standardised as self-report or interview administered.

Within the 21 tests, further selection was based on two considerations: (1) test development process (as well as initial evaluation) reported in a peer reviewed journal, and (2) behavioural scale contained in the test not made redundant by a scale contained in a later test covering the same (or very similar) set of specific methods of DSH. Nine tests were removed because of a lack of published information, and a further five tests were removed because of redundancy.

Six DSH tests (see Table 1) were therefore selected for the study, namely: Self-Injury Questionnaire Treatment Related (SIQTR) [34], Self-Injurious Thoughts and Behaviors Interview (SITBI) [26], Deliberate Self-Harm Inventory (DSHI) [16], Inventory of Statements About Self-Injury (ISAS) [6], Self-Harm Information Form (SHIF) [35], and Self-Harm Inventory (SHI) [21].

**Table 1:** Summary of psychometric qualities

**Short name of behavioural scale**	**Items**	**Sample**	**% DSH**	**Reliability**	**External validity**
SIQTR-5	A1, B1,C1, D1, E1	84, females, EDTP, av.age 24	30.4%^a^	α = 0.62^c^	Common SHI items r = 0.43 - 0.75^h^
SITBI-11	Q150: (1)-(11)	94, 77% females, MHU, av.age 17	68.1%^b^	Κ =1.0^e^	FASM, Κ = 1.0^e^; r = 0.99^g^
				ICC = .71^g^	
DSHI-16	Q1- Q16	150, 68% females, UG, age range 18 to 64	35%^b^	Φ = 0.68^e^, r = 0.92^f^	MHhx, r= 0.49^e^; BPO, r = 0.48^g^; SA r = 0.21^g^
				α =0.82^d^	
ISAS-12	Q1: (1)-(12)	235 UG (selected from 761 UG), 55% females, av.age 18	30.8%^b^	r = 0.85^g^	MSI-BPD, r = 0.37^g^; YRBS SI item, r = 0.38^g^; YRBS SA item, r = 0.28^g^
				α =0.84^d^	
SHI-16	Q1-Q16	290, 52% females, UG, av.age 20	68%^b^	Φ = 0.94^e^, r = 0.84^f^	NR
SHI-22	Q1–Q22	221, 90% females, MHU (4) PMC (104), EDTP/SATP (113), age range 17 to 63	71.9%^b^	α =0.80^d^†	DIB, r = 0.76^f^;
					PDQ-R, r = 0.73^f^

*EDTP *eating disorder treatment program, *SATP *substance abuse treatment program, *UG *under-graduates, *MHU *mental health unit, *PMC *primary medical care, α Coefficient Alpha, *Κ *Coefficient Kappa, Φ Coefficient Phi, *ICC *Intra Class Correlation, *MHhx* mental health history, *SI *suicide ideation, *SA *suicide attempt, *NR *not reported, *MSI-BPD *McLean Screening Instrument for Borderline Personality Disorder [36]; *YRBS* Youth Risk Behaviours Survey [37], *DIB* Diagnostic Interview for Borderlines [38], *PDQ-R* Personality Diagnostic Questionnaire Revised [39], *FASM* Functional Assessment of Self-Mutilation [40], *BPO* Borderline Personality Organization Scale [41], ^a ^one or more DSH behaviours in past month, ^b ^one or more DSH behaviours in lifetime, ^c ^items scored 0,1 for presence in last month, ^d ^items scored 0,1 for presence in lifetime, ^e^ dichotomous variable (lifetime presence) scored 0 (no items endorsed) and 1 (at least one item endorsed), ^f ^continuous variable (lifetime presence) scored as sum of 0,1 endorsements across all items, ^g ^continuous variable (lifetime frequency) scored as sum of frequency across all items, ^h^ continuous variable (frequency for each item) in last month, † [42] (107, 57% females, MHU, aged 18 to 65).

All six DSH tests contained additional items other than those related to methods of DSH. However, only the behavioural scales in each DSH test were relevant to the present study and, therefore, included (see Table 1). For clarity, the scales are referred to by the name of the full DSH test they come from while the number of items is indicative of the methods of DSH, rather than overall set of test items. For example, the ISAS-12 is extracted from the ISAS (total of 58 items) and contains 12 items covering specific methods of DSH.

Brief mention should be made of two well established DSH tests which were excluded from the present study. The first was the Self-Harm Behavior Questionnaire (SHBQ) [43] which was excluded due to the absence of a list of specific methods of DSH. The second was the Functional Assessment of Self-Mutilation (FASM) [44] which on examination showed the same set of specific DSH methods as the SITBI [26]. The FASM was therefore excluded in favour of the more recently published test.

### Changes to scale administration

Five of the DSH scales were self-report (using pencil and paper) while one (SITBI-11) was a structured interview. The SITBI-11 covers DSH behaviours with one question containing a list of 11 specific DSH behaviours presented orally (one behaviour at a time) to the interviewee. To maintain consistency with the other scales, the mode of administration of the SITBI-11 was changed to self-report (using pencil and paper).

All original versions of the DSH scales (except for the SHIF-16) contain an open response item at the end of the list of specific DSH behaviours. This allows respondents to indicate additional DSH acts (i.e., behaviours not included in the scale). In the present study, to avoid repetition the additional behaviour itemised under the ‘other’ option was applied once at the end of all the extracted behavioural scales. The ‘other’ item was not included in the scoring of any of the behavioural scales because the potential variability in this item would breach the requirement for scale standardisation and scale comparability.

### Range of DSH methods covered in each scale

In the absence of a comprehensive classification system of DSH behaviours [7], the scale items were grouped into three sets of behaviours based on a broad description of self-harm methods by Skegg [10]. The first group is DSH by self-injury with tissue damage, with common methods being cutting, scratching and burning [16]. The second group is DSH by highly dangerous methods, with common methods being drug overdose, self-strangulation, self-stabbing and swallowing harmful objects [45,46]. The third group is DSH by other self-harmful behaviours without visible injury, such as excessive exercising to hurt oneself [47], stopping medication [46], and deliberate recklessness (e.g., risk taking with cars to cause harm) [48]. It should be noted that the above groupings of behaviours are strictly within the dimension of method and no inference should be made about intent, outcome and lethality.

According to the groupings based on Skegg [10], all items contained in the SIQTR-5, SITBI-11 and DSHI-16 relate to DSH by self-injury with tissue damage. Most items in the ISAS-12 and SHIF-16 relate to DSH by self-injury with tissue damage, with one ISAS-12 item and three SHIF-16 items relating to DSH by highly dangerous methods. The SHI-22 contains six items related to DSH by self-injury with tissue damage, four items related to DSH by other self-harmful behaviours without visible injury, and one item related to DSH by highly dangerous methods. The SHI-22 also includes items covering indirectly self-harmful behaviours (4 items), maladaptive behaviours (4 items), psychological self-punishment (2 items), and motivation (1 item).

The instructions and item wording for all DSH behavioural scales orientate participants to respond to questions as intentional acts with the purpose of causing harm. All scales (except the SHI-22) also include instructions and item wording that orientate respondents to DSH without suicide intent. When combined with the range of methods covered in each scale, the construction of the SIQTR-5, SITBI-11 and DSHI-16 is consistent with the NSSI conceptualization of DSH. The ISAS-12 and SHIF-16 are generally consistent with NSSI although their inclusion of items related to DSH by highly dangerous methods (viz., swallowing dangerous substances, swallowing dangerous objects, and self-strangulation) is outside the range of methods associated with NSSI [14]. The instructions and item wording in the SHI-22 orientate respondents to DSH regardless of intent, and the range of DSH methods is consistent with a broad conceptualisation of DSH (e.g., [10]). The inclusion of non-DSH behaviours in the SHI-22 is consistent with a continuum of self-destructiveness [49].

### Response formats

The SHI-22 format is to endorse the lifetime presence for all items and to estimate the number of times during lifetime for most items. The SHIF-16 response format is to endorse lifetime presence, number of times in lifetime, and number of times in last 3 months. The SHIF-16 also allows respondents to report the age of onset and age of last occurrence. The DSHI-16 response format includes endorsement of lifetime presence, age of onset, number of times during lifetime, last occurrence, and number of years engaged in behaviour.

The primary SITBI-11 response format is lifetime endorsement, with additional questions that cover duration in years, number of acts in last 12 months, age of onset, and age of last act. For the ISAS-12, the primary response format is the frequency of specific methods of DSH in lifetime, followed by questions about the main form of self-harm including age of onset, date of most recent act, experience of pain, being alone, and time from urge to act.

Each of the specific methods of DSH in the SIQTR-5 items are assessed for recency with five response categories (a week, a month, several months, more than a year, never). When respondents indicate *a week* or *a month* they are directed to five more items covering body part, number of days in last month, number of times per day, frequency of pain, and duration of pain.

### Psychometric properties of selected scales

Evidence for the psychometric quality of the six DSH scales (as reported by scale developers) is summarised in Table 1. Four developers have reported Cronbach’s Alpha as a measure of internal consistency (which implies the intention to summate the item scores). Most developers have reported some evidence for test-retest reliability and external validity. None of the developers have reported evidence for unidimensionality.

### Participants

A sample of 568 young Australians participated in the study, comprising 440 females and 128 males, with an average age of 20.97 years (SD = 3.77). The sample included 350 university students (274 females, 76 males) (average age of 20.09 years, SD =2.87), 119 mental health patients (96 females, 23 males) (average age of 23.26 years, SD = 4.44), and 99 community members (70 females and 29 males) (average age of 21.32 years, SD = 3.41). A mixed sample was targeted in order to assess the psychometric properties of those scales across clinical and non-clinical populations and to provide comparability to the scale development samples (see Table 1).

The participants were recruited from the western suburbs of a large Australian city. All the mental health patients attended the same out-patient private mental health clinic, and responded to an information notice in the reception area. The university undergraduates were all enrolled in first year psychology and received course credit for their participation. The community members were recruited from several commercial and government workplaces who responded to information notices placed on staff notice boards.

English was the first language spoken by 72% of the participants, followed by Arabic (6%), Vietnamese (3%), Spanish (3%), Cantonese (2%), Greek (1%), Mandarin (1%) and Hindi (1%). The remaining 11% reported 26 other languages (each reported by less than 1% of the sample). The primary presentations reported by the mental health patients were depression and anxiety (26%), anxiety (24%), depression (15%), eating disorder (14%), alcohol and other drugs (7%), and other conditions (14%) including relationship difficulties and situational crises.

### Procedure

The ISAS-12, SHIF-16, and SHI-22 were administered to 332 participants (called Sample 1). The SIQTR-5, SITBI-11, DSHI-16, SHIF-16, and SHI-22 were administered to 236 participants (called Sample 2). Sample 1 (58.5% of all participants) comprised 200 undergraduates (166 females and 34 males), 65 mental health patients (54 females and 11 males), and 67 community members (46 females and 21 males). Sample 2 (41.5% of all participants) comprised 150 undergraduates (110 females and 40 males), 54 mental health patients (42 females and 12 males), and 32 community members (25 females and 7 males).

Responses were scored 0 (behaviour never engaged in during lifetime) and 1 (behaviour engaged in at least once during lifetime). This scoring method is consistent with the most common scoring method used by the scale developers when reporting endorsement rates and Cronbach’s Alpha (which implies the intention to summate the item scores) (see Table 1). Further, this scoring method is a common procedure for forming a DSH total score by summing the number of methods of DSH over a person’s lifetime (e.g., [21]). Provided the specific DSH methods in each scale are hierarchically ordered, total scores may quantify the degree to which respondents have progressed to the more severe end of a DSH latent construct [15] in response to increasing levels of psychological distress or continued failed coping [50].

Both samples contained the SHI-22 and SHIF-16 to provide a common item equating structure to calibrate the total set of items onto a single underlying metric, via Rasch analysis [51]. The SHI-22 and SHIF-16 were selected as the common measures because they contain the most expansive sets of items with respect to number of items and methods of DSH.

The scales were reproduced in a printed test booklet. Ethical approval was granted by the University of Western Sydney Research Ethics Committee. Participants’ informed consent was obtained in accordance with the Declaration of Helsinki.

### Analysis

The Rasch model tests whether or not there is a quantitative structure underlying the response to items, such that the attributes of Additive Conjoint Measurement can be satisfied, and an interval-scale transformation of the raw score obtained [52,53]. The Rasch analysis was conducted using RUMM 2030 software [54]. The items in each scale were tested for appropriate stochastic ordering (fit) and local independence assumptions (response dependence and unidimensionality) [55]. Further tests were also undertaken on the invariance of the scales (Differential Item Functioning or DIF) across defined’person factors’ including age (18 to 19 years vs. 20 years and above) and gender (females vs. males). A number of chi-square and residual fit statistics were used to test if the data satisfy model expectations, and ideal values of these are presented in the last row of Table 2. It is accepted practice to evaluate overall fit to the Rasch model by the use of Bonferroni adjusted *p* values (i.e., 0.05 divided by the number of items) [30], a conservative [56] yet widely accepted correction for repeated statistical tests [57].

**Table 2:** Results of Rasch analyses

**Analysis**	**Overall model fit**	**Item fit residual mean (SD)**	**Person fit residual mean (SD)**	**% significant t-tests**	**Coefficient Alpha (Grading)**	**Person locations**
SIQTR-5						
Initial fit	*χ*^2 ^= 6.639 (d f= 5), *p *= .249	-0.786 (0.922)	-0.136 (0.548)	na	0.705	M = -1.238
					Moderate	SD = 1.131
Final fit	*χ*^2 ^= 7.320 (d f= 6), *p *= .292	-0.503 (0.928)	-0.069 (0.583)	na	na	M = -1.324
						SD = 1.161
SITBI-11						
Initial fit	*χ*^2 ^= 52.463 (d f= 22), *p *= .0003	-0.271 (1.384)	-0.186 (0.649)	0.42%	0.716	M = -1.662
					Fair	SD = 1.295
Final fit	*χ*^2 ^= 46.978 (d f= 26), *p *= .007	-0.254 (1.162)	-0.202 (0.649)	na	na	M = -1.629
						SD = 1.325
DSHI-16						
Initial fit	*χ*^2 ^= 33.957 (d f= 32), *p *= .373	-0.278 (1.165)	-0.185 (0.351)	1.69%	0.783	M = -3.117
					Fair	SD = 1.159
Final fit	*χ*^2 ^= 30.790 (d f= 24), *p *= .160	-0.127(1.209)	-0.254 (0.560)	na	na	M = -2.495
						SD = 1.136
ISAS-12						
Initial fit	*χ*^2 ^= 38.299 (d f= 24), *p *= .032	-0.278 (0.962)	-0.123 (0.713)	1.81%	0.826	M = -1.872
					Fair	SD = 1.358
Final fit	*χ*^2 ^= 23.441 (d f= 18), *p *= .174	-0.171 (0.883)	-0.164 (0.700)	na	na	M = -1.802
						SD = 1.185
SHIF-16						
Initial fit	*χ*^2 ^= 150.982 (d f= 64), *p *= .000	-0.731(1.951)	-0.210 (0.454)	1.6%	0.794	M = -2.484
					Fair	SD = 1.195
Final fit	*χ*^2 ^= 70.304 (d f= 46), *p *= .012	-0.708 (1.174)	-0.290 (0.676)	na	na	M = -1.828
						SD = 1.012
SHI-22						
Initial fit	*χ*^2 ^= 289.355 (d f= 154), *p *= .000	-0.537 (1.740)	-0.179 (0.712)	5.50% (3.7-7.3)	0.814	M = -2.051
					Fair	SD = 1.280
Final fit	*χ*^2 ^= 201.992 (df =153), *p *= .005	-0.460 (1.220)	-0.215 (0.764)	na	na	M = -1.944
						SD = 1.201
Ideal Values	Probabilities greater than Bonferroni adjusted *p *values (i.e., 0.05 divided by the number of significance tests)	Mean = 0.0	Mean = 0.0	Less than 5%	Fair or better^a^	M = 0.0
		(SD = 1.0)	(SD = 1.0)			

^a^Gradings based on Ponterotto & Ruckdeschel [58] and take into account number of items and sample sizes.

RUMM 2030 also provides a post-hoc test of the unidimensionality of the items, given Rasch analysis is a confirmatory procedure. This is conducted with a principal component analysis (PCA) on the standardised residuals for the items to form one sub-test containing items with positive loadings and another sub-test containing items with negative loadings. The person ability estimates obtained from each sub-test are then compared using independent t-tests [59]. Unidimensionality is indicated when very few of the t-tests (less than 5%) are significant.

The unidimensionality of tests (and item fit statistics) can be adversely impacted by local response dependency. This occurs when the response on one item influences the response on another item. Under these circumstances items are combined into sub-tests so that local dependency within the test items is absorbed [60]. Thus, dichotomous items are clustered to make polytomous items.

The Rasch model has no distributional assumptions, and does not require any form of representative sample. Rather, for calibration purposes, a uniform distribution is useful in contributing to an equal degree of precision of item estimates across the metric, but is not a requirement.

## Results

### Rates of DSH

Overall rates of DSH for the present study are based on the reporting of at least one specific method of DSH within each of the three broad groupings of DSH methods provided by Skegg [10]. The selected items for each grouping are from the common scales (SHIF-16 and SHI −22) in order to provide rates for the full sample (n = 568). For completeness, the rate of endorsement for the attempted suicide item in the SHI-22 is also reported, although this is not a specific method of DSH.

The rates of DSH showed a strong trend by grouping of methods and for suicide attempts, with 11.1% reporting highly dangerous methods (not necessarily wanting to die), 13.9% reporting a suicide attempt, 39.4% reporting self-injury with tissue damage, and 45.6% reporting other self-harmful behaviours without visible injury. Females were much more likely to report dangerous methods (12.0% in females vs 7.8% in males), suicide attempts (16.4% in females vs 5.5% in males), and tissue damage (42.9% in females vs 27.3% in males). The gender rates for DSH by other self-harmful behaviours without visual injury were 47.6% in males and 45.0% in females.

There was also a strong trend by sub-sample, with mental health patients reporting the highest levels for all three groupings of DSH methods and for suicide attempts, followed by undergraduates and community members. Highly dangerous methods were reported by 17.6% of patients, 11.4% of undergraduates, and 1.0% of community members. Self-injuries with tissue damage methods were reported by 55.5% of patients, 37.4% of undergraduates, and 27.3% of community members. Other self-harmful behaviours without visible injury were reported by 66.4% of patients, 42.0% of undergraduates, and 33.3% of community members. Suicide attempts were reported by 36.1% of patients, 8.6% of undergraduates, and 6.1% of community members.

### Initial model fit

The results of the Rasch analyses of the six scales are reported in Table 2. The original versions of the SIQTR-5, DSHI-16, and ISAS-12 showed adequate fit to the Rasch model (based on item-trait interaction). The original versions of the SITBI-11, SHIF-16 and SHI-22 showed a lack of fit to the Rasch model (based on item-trait interaction), noting the application of Bonferroni adjusted *p* values (i.e., 0.05 divided by number of significance tests).

### Local response dependency

**Figure 1:**
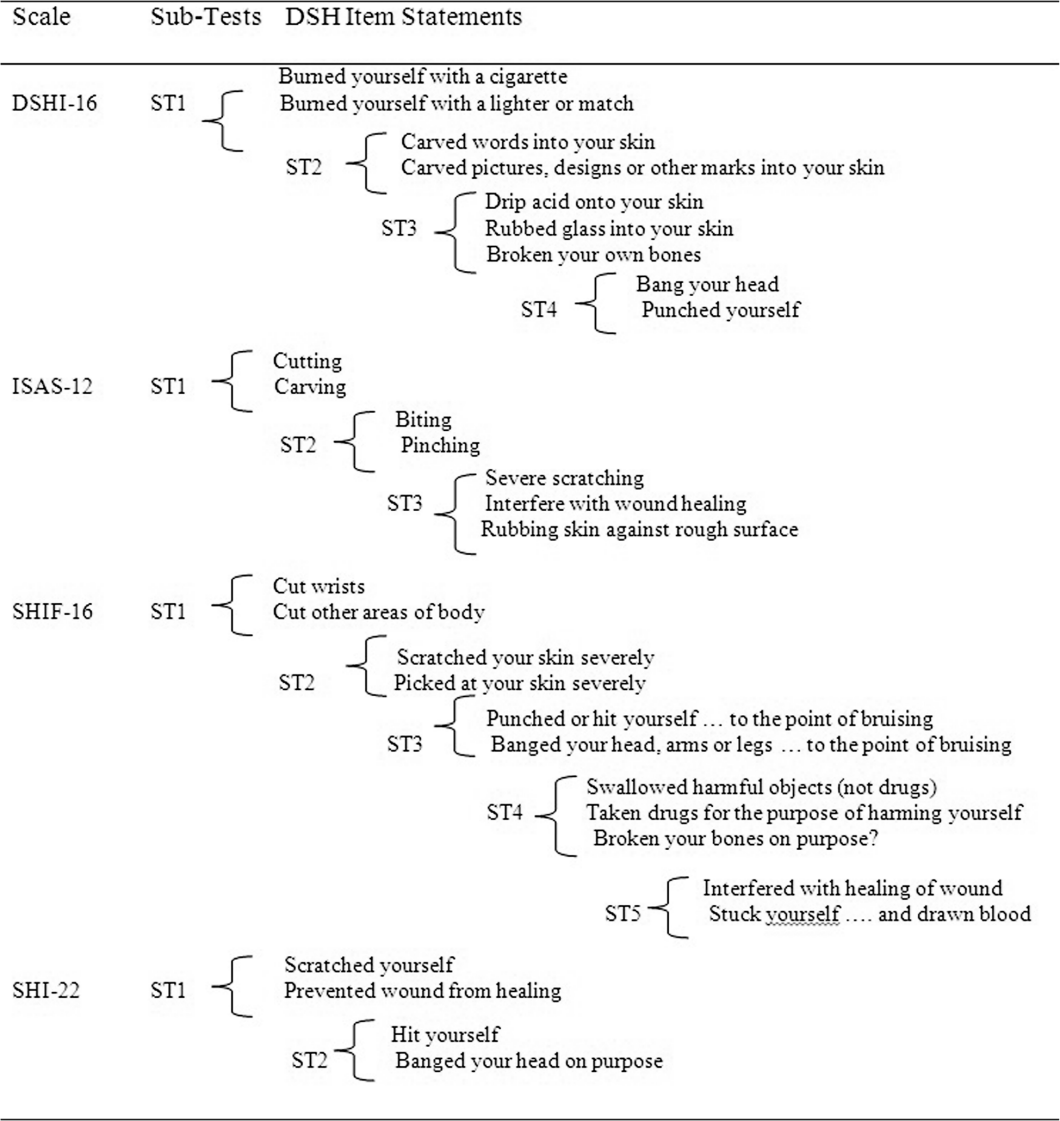
DSH items with local response dependency.

There was no local response dependency for SIQTR-5 and SITBI-11. However, items in the other four scales exhibited local response dependency, using values for residual correlations 0.20 above the average of all residual correlations. Local response dependency in all four scales was resolved by the use of sub-tests (see Figure 1).

There are three patterns of response dependencies shown in Figure 1. First, some items are conditional on each other, for example, SHIF-16 Items 11 (cut wrists) and SHIF-16 Item 12 (cut other areas of body). Second, some items are likely to have the same (or almost the same) meaning to respondents, for example, DSHI-16 Item 2 (burned yourself with a cigarette) and DSHI-16 Item 3 (burned yourself with a lighter or match). Third, some items are highly inter-related, for example, ISAS-12 Item 7 (severe scratching), ISAS-12 Item 9 (interfere with wound healing), and ISAS-12 Item 10 (rubbing skin against a rough surface).

### Differential Item Functioning (DIF)

Four scales (SIQTR-5, DSHI-16, ISAS-12 and SHIF-16) contained items with significant uniform DIF for gender, with the use of Bonferroni adjusted *p* values (0.05 divided by number of significance tests). The items were SIQTR-5 Item 3 (cut yourself; females > males), DSHI-16 Item 1 (cut yourself; females > males), ISAS-12 Item 1 (cutting; females > males), SHIF-16 Item 11 (cutting wrists; females > males), and SHIF-16 Item 6 (punched or hit yourself; males > females).

Two scales (SITBI-11 and SHI-22) contained items with significant uniform DIF for both person factors (gender and age), again with the use of Bonferroni adjusted *p* values (0.05 divided by number of significance tests). In the SITBI-11, Item 1 (cut or carved skin) showed DIF for gender (females > males) and Item 8 (bite yourself) showed DIF for age (18 to 19 years > 20 years and above). In the SHI-22, Item 2 (cut; females > males), Item 7 (driven recklessly; males > females), and Item 17 (lost job on purpose; males > females) showed DIF for gender. Also in the SHI-22, Item 4 (hit; 18 to 19 years > 20 years and over), Item 7 (driven recklessly; 20 years and over >18 to 19 years), and Item 11 (been promiscuous; 20 years and over >18 to 19 years) showed DIF for age.

### Misfitting Items

Across the six scales, six items exhibited individual item fit residuals greater than +/− 2.5. Except for SHIF-16 Item 2 (bitten fingernails to cause bleeding or pain), individual item misfit was resolved by the adjustments made for local response dependency and DIF. The source of the misfit for SHIF-16 Item 2 was lack of discrimination, that is, the probability of response was the same across all overall levels of DSH. The lack of discrimination was specific to the clinical sample.

### Final model fit

Scale modifications (as necessary to fully meet all the assumptions of the Rasch measurement model) included formation of sub-tests to deal with local response dependency (all scales except SIQTR-5 and SITB-11I), item splitting to resolve differential item functioning (all scales), and the deletion of one misfitting item (bitten fingernails to cause bleeding or pain) in the SHIF-16. The final fit statistics for all scales (see Table 2) indicated adequate fit to the Rasch model, noting the application of Bonferroni adjusted *p* values (i.e., 0.05 divided by number of significance tests).

### PCA tests

The PCA test of unidimensionality was not conducted for the SIQTR-5 because of the small number of items. When conducted for all other scales, the PCA tests supported strict unidimensionality, using the 5% criteria (see Table 2). It should be noted that the PCA test for the SHI-22 showed 5.50% of the t-tests to be significant, but the lower bound of the confidence interval (CI: 3.7 – 7.3%) was below 5%. PCA tests were not conducted for final models as they all included at least one split item (as necessary to resolve DIF) and so contained structural missing cases.

### Item hierarchies

For each scale, items were ordered according to their locations (in logits) on the latent construct from most easy to endorse (with negative logit values) to most difficult to endorse (with positive logit values). The items located at the top and bottom of the item hierarchy for each scale are now listed, with location and standard error given in brackets. In order to allow comparison of locations across scales, the 82 items were calibrated on the same metric.

The SIQTR-5 hierarchy ranged from cutting (−1.278, 0.169) to burning (0.086, 0.222). The DSHI-16 hierarchy ranged from cutting (−1.217, 0.170) to dripping acid on skin (4.735, 1.475). The ISAS-12 hierarchy ranged from banging or hitting self (−1.351, 0.142) to sticking self with needles (1.015, 0.242). The SHIF-16 hierarchy ranged from interfering with wound (−1.647, 0.106) to breaking bones (3.291, 0.497). The SITBI-11 hierarchy ranged from pick wounds (−2.383, 0.156) to erased skin (1.543, 0.352). The SHI-22 hierarchy (including DSH non-DSH methods) ranged from torture yourself with self-defeating thoughts (psychological self-punishment) (−2.068, 0.103) to abuse laxatives (indirect self-harm) (1.476, 0.223).

### Targeting

The samples for each scale (as a whole) exhibited a lower level of DSH than the average level of DSH measured by the scale, as indicated by the negative values for the mean person locations (ranging from −3.117 for the DSHI-16 to −1.238 for the SIQTR-5) (see Table 2). This finding is reflected in the endorsement rates (at least one behaviour reported by participants) for each scale. The DSHI-16 and SIQTR-5 (easiest items related to cutting behaviours) showed endorsement rates of 48% and 51%, respectively. The ISAS-12 (easiest items related to banging behaviours) showed an endorsement rate of 60%. The SHIF-16 and SITBI-11 (easiest items related to wound picking) showed endorsement rates of 65% and 76%, respectively. The SHI-22 (easiest item was a non-DSH item related to psychological self-punishment) showed an endorsement rate of 79%.

### Reliability

The grading of the reliability estimates in Table 2 are based on Ponterotto & Ruckdeschel [58] and take into account number of items and sample sizes. All gradings were rated as fair or moderate, with the obtained Cronbach’s Alpha values ranging from 0.71 (SIQTR-5) to 0.83 (ISAS-12). Cronbach’s Alpha estimates are not provided for the final models that included at least one split item. This is because the splitting of items (as necessary to resolve DIF) results in structural missing cases.

## Discussion

The first aim of this study was to evaluate the psychometric properties of six DSH behaviours scales. According to the stringent post-hoc tests provided by the Rasch measurement model [29], there is support for the unidimensionality of the sets of items contained within each of the scales.

The fit to the Rasch model confirms the hierarchical ordering of the specific methods of DSH contained in each scale [32], and justifies the counting of different methods (scored 0,1 for lifetime presence) in each scale to form a total DSH score. Such a total score can be used to order people on a DSH latent construct, with high scores indicating a progression to more severe methods. This finding supports the causal models of DSH that incorporate a mechanism to explain an escalation of behaviours (e.g., [50]), and validates the tentative ordering of specific methods reported in the literature as based on clinical experience and/or conceptual labeling [10,15].

The fit to the Rasch model also provides researchers with the opportunity to convert ordinal raw scores into an interval scale estimate of the latent trait [51], as is appropriate when applying parametric statistical procedures [30]. Some minor modifications to the six DSH scales were required to fully meet the assumptions of the Rasch model, namely, the formation of sub-tests to deal with local response dependency (all scales except SIQTR and SITBI), item splitting to resolve differential item functioning (all scales), and item deletion to deal with one grossly misfitting item (nail biting to cause bleeding or pain) in SHIF. However, all the above adjustments can be conducted within the computational procedures (e.g., RUMM2030) and do not require any changes to the administration procedures.

The second aim of this study was to examine the applicability of the scales across age and gender by the use of DIF analyses (also called item bias). With respect to gender, cutting behaviours are more likely to be endorsed by females while self-hitting behaviours are more likely to be endorsed by males. With respect to age, the self-biting items are more likely to be endorsed by younger persons. There is also evidence that some methods of DSH involving deliberate recklessness to cause harm are more likely to be endorsed by older persons. The gender and age biases may be clinically informative, and possibly lead to a better understanding of the differential prevalence rates [11].

The third aim of this study was to inform the item hierarchies within each scale. Although the hierarchies in each scale are probabilistic, a person who endorses an item reflecting higher order self-harm behaviour (such as SHIF Item 15 broken bones on purpose) will have endorsed some other items in the scale, and certainly the items reflecting lower order behaviours (such as SHIF Item 1 interfered with wound healing), where there would be a 0.95+ probability of affirmation. The item hierarchies, therefore, may provide clinically significant information, as supported by recent longitudinal evidence that future DSH is best predicted by the range of past DSH behaviours [19].

For each scale, the prevalence rate of DSH (based on the endorsement of at least one specific method of DSH) is influenced by the item hierarchy. That is, scales with a hierarchy that commences with easy to endorse methods such wound picking (SITBI, SHIF) will bring people into the DSH classification sooner than scales with a hierarchy that commences at less easy to endorse methods such as banging or hitting self (ISAS) and cutting (DSHI and SIQTR). This is particularly evident in the SHI item hierarchy which commences with non-DSH items related to psychological self-punishment and indirect self-harm, that may be more normative than DSH behaviours [10]. In sum, the nature of the item hierarchies in each scale may assist in the understanding of different prevalence rates across studies, and may inform the debate on the relative merits of single item versus multiple method scales of DSH [61].

The fourth aim of this study was to inform scale selection for clinicians and researchers. Prior to the present study, little was known about the unidimensionality of DSH behavioural scales despite it being an accepted standard for scale selection [25]. Based on the evidence for unidimensionality provided in the present study, in combination with the evidence for reliability and external validity provided by scale developers, clinicians and researchers can be confident of the overall psychometric quality of the six scales.

Given the overall adequacy of all six scales, clinicians and researchers are recommended to select the scale that best matches their adopted definition of DSH. The DSHI, SIQTR, SITBI are most relevant to a narrow conceptualisation of DSH methods (e.g., NSSI; [9]). The ISAS and SHIF are most appropriate to a broader conceptualisation of DSH methods that combines tissue damage methods (such as cutting and burning) with highly dangerous methods (such as strangulation and swallowing dangerous objects) (e.g., [10]). The SHI is most suitable for the measurement of a wide spectrum conceptualisation of DSH methods (e.g., [62]) and may be of particular value for the measurement of a broad continuum of self-destructive behaviours [49] in specific clinical populations, such as borderline personality disorder [63].

The present study is not without limitations. First, the administration procedures for the SITBI were adapted from interview to self-report to maintain consistency across the scales, although this did not alter scale scoring. Second, the study lacked diagnostic confirmation of the clinical sample, although they were recruited from clients attending a psychology clinic on referral from primary care physicians. Third, the study would have benefited from the inclusion of younger participants to provide coverage of the likely age of onset (10–14 years) to the peak period of DSH in adults (18 to 30 years) [11]. Fourth, there was a dominance of female participants, although the gender ratio is reflective of the DSH gender prevalence [11], and is similar to the proportions in the scale development samples (see Table 1). Fifth, the psychometric qualities of the behavioural scales are based on items scored 0,1 for lifetime presence rather than current episodes. Future studies should build on the findings of the current study by examining the unidimensionality of DSH scales in younger populations, across specific psychopathology diagnoses, and covering frequency of behaviours as well as range of methods (with item scoring for both lifetime presence and recent episodes).

## Conclusions

This study provides a comprehensive evaluation of the psychometric properties of six commonly used DSH behavioural scales across a large sample representative of student, clinical and community young people. Importantly, it demonstrates that these scales are psychometrically sound as examined against the stringent standards provided by the Rasch measurement model. The findings of item bias and local response dependency may inform scale interpretation at both clinical and research levels. Further, the findings support the use of behavioural items to measure a DSH construct, and the hierarchy of behaviours in each scale may inform the risk of future DSH. Importantly, this study shows that similar levels of psychometric quality can be expected from the six scales even though they range in content from visible tissue damage behaviours to a broad continuum of self-destructive behaviours. Clinicians and researchers, therefore, may select a scale that is most congruent with their conceptualization of DSH.

## Competing interests

The authors declare that they have no competing interests.

## Authors' contributions

SL and TM participated in the study design and coordination. SL, TM and AT performed the statistical analysis. SL and TM drafted the manuscript. All authors contributed to and approved the final manuscript.

## Pre-publication history

The pre-publication history for this paper can be accessed here:

http://www.biomedcentral.com/1471-244X/13/4/prepub
